# An easy to implement logic analyzer for long-term precise measurements

**DOI:** 10.1016/j.ohx.2020.e00164

**Published:** 2020-12-15

**Authors:** Alexey M. Romanov

**Affiliations:** MIREA – Russian Technological University, Russia

**Keywords:** Logic analyzer, Jitter, Long-term measurements, Field-programmable gate array

## Abstract

Most of market-available logic analyzers are designed for hardware debug purposes and cannot record continuous measurement in long-term while in different fields of scientific research it is necessary to make data acquisition within small periods (less then 1 ms) during several hours or even days. The common example is real-time communication worst-case jitter analysis. This paper introduces an easy to implement approach how to create a logic analyzer for such kind of task on a basis of a low-cost Field-Programmable Gate Array (FPGA) kit and a personal computer. The Author provides both sample FPGA design files compatible with an open-source toolchain and the approach how to collect data using standard software and Octave scripts to post-process the experimental result. Following the Author’s guidelines even with minimal knowledge in FPGA design makes it easy to modify the introduced hardware for specific laboratory team needs.

## Graphical abstract

**Figure:**
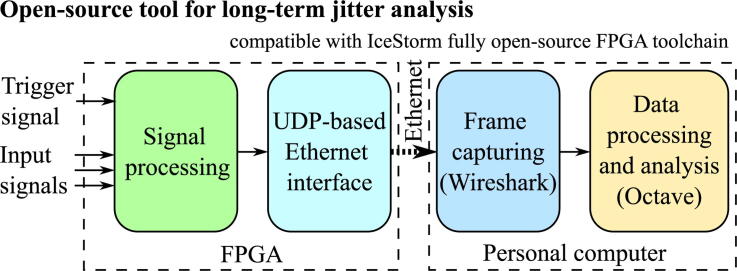

Specifications table:

**Table:** 

Hardware name	Logic analyzer for long-term jitter monitoring

Subject area	• Engineering and Material Science • Educational Tools and Open Source Alternatives to Existing Infrastructure

Hardware type	• Field measurements and sensors • Electrical engineering and computer science

Open source license	Creative Commons Attribution-ShareAlike license.

Cost of hardware	61.5–265 USD depending on number of channels and chosen Field-Programmable Gate Array platform.

Source file repository	http://doi.org/10.5281/zenodo.4034301https://github.com/amromanov/open_la

## 1 Hardware in context

Real-time computing and communication are key technologies required for modern automation control [1]. Lots of research teams around the world develop different solutions to improve real-time parameters of software [2], [3], [4], [5], wired [6], [7], [8], [9] and wireless [10], [11], [12] communication, the main of which is the worst-case clock jitter.

There are different ways to measure the jitter during experimental studies. In real-time software design the jitter is often measured by a special precise hardware timer which works independently from the central processing unit. In communication tasks it is usually necessary to measure the clock jitter not for each single device, but between the clocks or different devices and a chosen master clock. For the wired real-time networks there are special proprietary devices designed for jitter measurement (such as B&R X20ET8819 for Ethernet POWERLINK [6]), but such kind of devices can work only in compliance with specific communication protocols, thus restricting their use in scientific research.

**Fig. 1:**
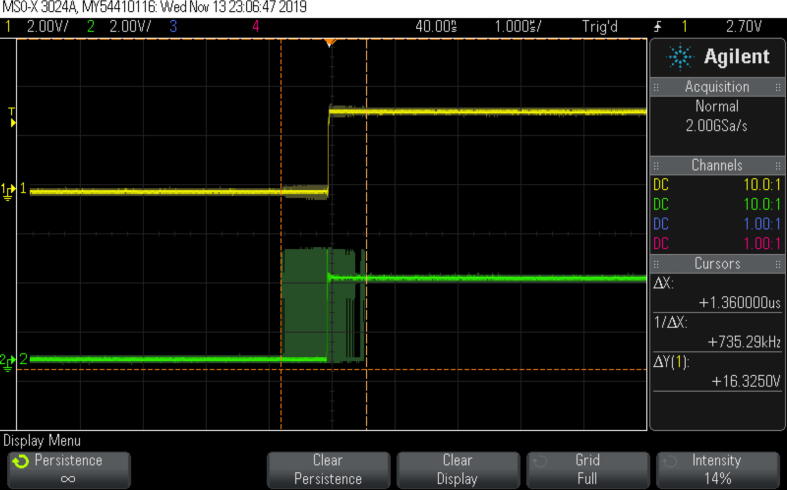
An example of worst-case device-device clock jitter measurements using oscilloscope.

The most general way to evaluate jitter in this case is to generate square-wave signals synchronized to the internal clocks of the devices and to monitor them with a digital oscilloscope triggered by a square-wave based on a master clock [13]. If a digital oscilloscope is switched into the Persistence mode, then the left-most and right-most edges of the overlapped square-wave recorded during the measurement period will show the range ΔX corresponding to maximum device-device clock jitter (Fig. 1).

Practically, the method described above suffers from several major drawbacks:

• Digital oscilloscopes are expensive and complex devices which makes their usage more complicated in areas with potentially aggressive environment (outdoor, inside thermal cameras, etc.).
• Digital oscilloscopes have few independent channels (generally 2–4).
• The above described method makes possible to measure only absolute values of the worst-case jitter while statistical parameters of jitter distribution using digital oscilloscopes in long terms still remains a challenge.

A logic analyzer can be used as an alternative. Unlike the digital oscilloscope, the logic analyzer does not include an analogue to digital converters, and it measures only discrete signals with a standardized voltage levels. They are generally cheaper than oscilloscopes and are equipped with much more channels (usually about 16–32). Some logic analyzers are designed as a part of a digital oscilloscope (for example Keysight MSO-X 3024A [14]), while others can serve as a totally independent device (Zeroplus Logic Cube [15] or similar).

For the last decade several open-hardware logic analyzers were introduced. One of the oldest known open-hardware logic analyzers is MiniLA [16]. It is designed on a basis of Complex Programmable Logic Device (CPLD) Xilinx XC95288XL and uses Line Print Terminal (LPT) or Universal Serial Bus (USB) interfaces to communicate with a personal computer (PC). According to the SourceForge information, the projects have not been updated since December 2012. Probably, the most well-known open-hardware logic analyzer is SUMP [17]. It is built on a basis of Xilinx Spartan-3 Field-Programmable Gate Array (FPGA), which communicates to a Java client on a PC using 115200 bps Universal Asynchronous Receiver-Transmitter (UART) interface (or USB through USB-UART adapter). SUMP2 is also based on FPGA, but it has a more complex architecture compared to SUMP making it more similar to proprietary FPGA vendor solutions, such as Xilinx ChipScope and Altera SignalTap [18]. Also SUMP2 has PC software written on python instead of Java. A very similar solution compatible with original SUMP software was designed by the team from the University of Alabama in Huntsville [19]. Finally, one of the most advanced open-hardware logic analyzers, BitHound, was designed by the team from ETH Zurich [20]. It can process up to 16 channels at 400 MHz sampling rate, features 128 MB sample memory and is equipped with the 100 Mbps Ethernet interface. The last feature not only reduces time required to download samples into PC, but also makes possible to place the logic analyzer up to 100 m from PC, which makes it crucial for field experiments.

**Fig. 2:**
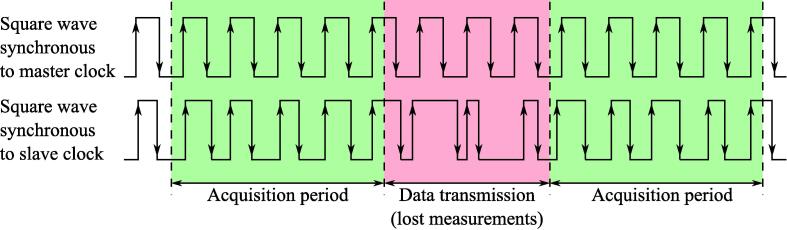
Edge missing during long terms jitter timing analysis using logic analyzer.

Unfortunately, the above described solutions, including proprietary commercial devices, have the same drawback, which makes them non applicable for the long-term jitter analysis. All of them were designed mainly for hardware design and debugging purposes, when developer records short sequences and then manually analyzes them. The operation sequence of these devices can be basically described as “Wait for Trigger” – “Measure” – “Buffer” – “Transmit”. Unfortunately, even quite large size of buffered data in advanced models does not guarantee that none of edges will be missed during data transmission (Fig. 2). Therefore, these missing edges can result in/lead to faulty-lower jitter estimation. This problem becomes crucial for the devices based on low-speed interfaces, such as LPT, UART and USB-UART (for example Zeroplus Logic cube analyzer can record only one of two 50μs sequences per second), but is real-time continuous acquisition is usually badly supported by software even in the Ethernet-based devices. Another result of the debug-oriented usage model is the fact that modern logic analyzers record signal levels with a fixed sample time (usually 5–20 ns), which is enough when it is needed to analyze signal sequences with duration up to hundreds of milliseconds, but is not enough for long term monitoring requiring experiments to last several hours or even days. In this case, the amount of stored data will exceed dozen gigabytes per experiment, thus compicating its processing even worse. On the other hand, making rare measurements will complicate the understanding circumstances that cause jitter. The good solution would be to use dynamic acquisition rate, which will become higher around points of interest with the jitter beyond some limit, but to the best of the Author’s knowledge, such kind of mode is not supported in above described devices.

The design proposed in this paper aims to overcome the problems stated and provides an easy to implement solution for long-term jitter monitoring.

## 2 Hardware description

The idea of the proposed device came to the Author’s mind in 2018, while he was verifying real-time communication modules on Barneo Ice Camp near the North Pole. It was necessary to make long term jitter measurements for outdoor-mounted devices. There were FPGA kits that were taken as spare part. One should also take into consideration that there was no access to laboratory equipment, such as oscilloscopes or logic analyzers, as well as Internet access to download any open-source logic analyzers described in the previous sections. The goal was to design a robust solution, which will enable one to record measurements during several weeks and simultaneously reduce time for its software and hardware debugging as much as possible/to a maximum rate. Then the proposed approach was successfully used/applied to several times in other projects dedicated to wired [21] and wireless real-time communications [22]. Finally, now it is well commented and documented to be ready for sharing with scientific community.

**Fig. 3:**
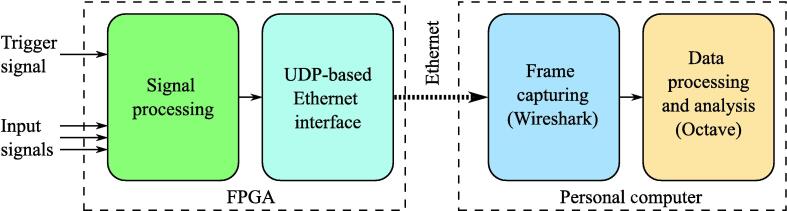
A general structure of the proposed solution.

A general structure of the proposed solution is shown in Fig. 3.

The first distinction from the widely used solutions is that it does not have any buffers to store Samples. Instead, it processes the input signals by FPGA in real-time and transmits them using 100 Mbps Ethernet directly to PC. As it has been mentioned above, buffering does not provide any real benefits in long term monitoring because, in case the interface bandwidth is not big enough, then any buffers will overflow anyway. At the same time, bufferless processing significantly simplifies overall design dramatically reducing the time required to debug new signal processing functions, thus making it possible to implement proposed logic analyzer on a wide range of low-cost FPGAs.

The second key difference is that, unlike special software on PC, the combination of Wireshark and Octave tools is used. While other logic analyzers were designed as standalone self-contained devices with wide but predefined functionality, the proposed solution is considered as a starting point for a rapid prototyping of a task-specific tool. Following this paradigm Wireshark gives robust functionality to capture all the traffic from FPGA with regard to network settings and Octave provides flexible tools to process and display captured data. A strict separation between capturing and data processing while using two independent tools has a significant benefit: it ensures the safety of important experimental data and prevents from its possible loss caused by the faulty processing software. The other benefit is that researchers can easily and separately change both data capturing and data processing tools without introducing any changes in hardware or in the method in general. For instance, Wireshark can be easily changed to dumpcat, and Octave – to MATLAB or Python. All the necessary scripts can be adopted from the ones provided with this paper in no time.

Speaking of the implementation aspects of the proposed tool, one can point out that the overall design is extremely modular, and cross-platform makes it easy to modify it or port it to new FPGA architectures even for the researchers with minimum Hardware Description Language (HDL) design experience.

**Fig. 4:**
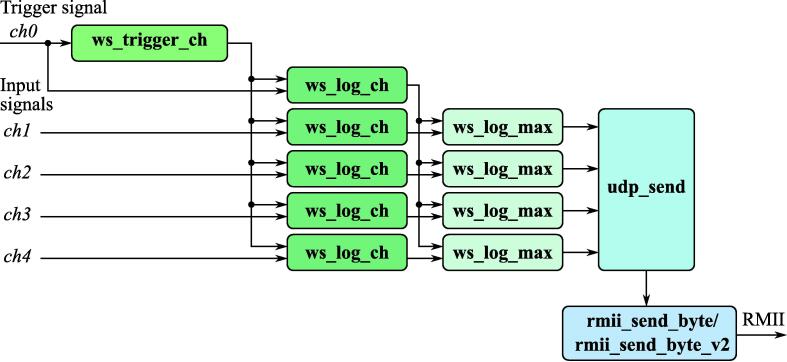
A structure of the FPGA design for the reference 4-channel logic analyzer.

The proposed FPGA design contains 5 main modules, which can be combined in different ways to achieve the desired level of performance and functionality. A structure of the FPGA design for a reference device implemented in this paper is shown in Fig. 4.

**Fig. 5:**
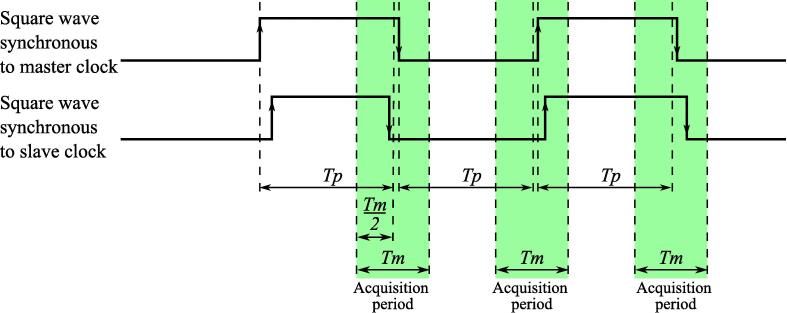
Data acquisition timing diagram provided by **ws**_**trigger**_**ch** IP core.

**ws**_**trigger**_**ch** Intellectual Property (IP) core detects edge on a master channel and creates a mutual time source for another channel in a measurement window (Fig. 5). Generally, the measurement window has its center after *Tp* clock cycles from the last edge of the master channel and has width *Tm* clock cycles. In the long-term jitter analysis application *Tp* is typically a value close to period of a square wave synchronous to master clock and *Tm* is at least two times higher than the desired worst-case jitter.

**ws**_**log**_**ch** IP core timestamps edges using time source provided by **ws**_**trigger**_**ch** and should be used for all signals, including the one, which feeds **ws**_**trigger**_**ch**. Both **ws**_**log**_**ch** and **ws**_**trigger**_**ch** have an internal synchronizer and a configurable filter, which makes possible to safely connect their input directly to FPGA input buffers.

**ws**_**log**_**max** IP core takes output values of **ws**_**log**_**ch** blocks and evaluates the difference between edge timestamps for signal corresponding to master clock and slave ones, which is then considered as the immediate jitter value. This IP core has also a dynamically changing output rate, which provides highly detailed output with jitter being beyond the defined limit and returns the worst-case jitter which occurred in the last $2^{\mathrm{Npr}}$ measurements every $2^{\mathrm{Npr}}$ measurements, while the jitter is low. Moreover, those limits can be set individually for each channel. This makes possible to tune the amount of the data collected during the experiment regardless of the frequency of measurements. **ws**_**log**_**max** is the example of an application-specific IP core, which can be replaced by the other ones while performing tasks different from jitter analysis in multiple device synchronization.

**udp**_**send** is a fully hardware generator of User Datagram Protocol (UDP) frames. It is designed in such a way, so that its parameters and payload can be easily customized from the top module, while the IP core will require no additional modifications.

**rmii**_**send**_**byte** and **rmii**_**send**_**byte**_**v2** implement Reduced Media Interface (RMII) communication between FPGA and Ethernet physical layer integrated circuit (PHY). **rmii**_**send**_**byte** should be used in designs clocked at 100 MHz, while **rmii**_**send**_**byte**_**v2** is designed to operate with 50 MHz clock. This makes possible to implement the proposed logic analyzer on ultra low-cost FPGA families, such as Lattiec iCE40, which cannot run complex designs at 100 MHz frequency due to internal latencies.

All the designed IP cores work in a single clock domain. They are written on plain Verilog, do not include soft processors, multipliers or RAM blocks and do not require any proprietary modules from FPGA vendors, even Phase-Locked Loop (PLL). This makes them fully compatible with most FPGAs on the market including Intel, Xilinx, Lattice, etc. Moreover, the present paper demonstrates the way to build and program proposed design with fully open-source FPGA toolchain and without any additional proprietary software. Such flexibility and openness are the key advantages of the proposed architecture.

**Fig. 6:**
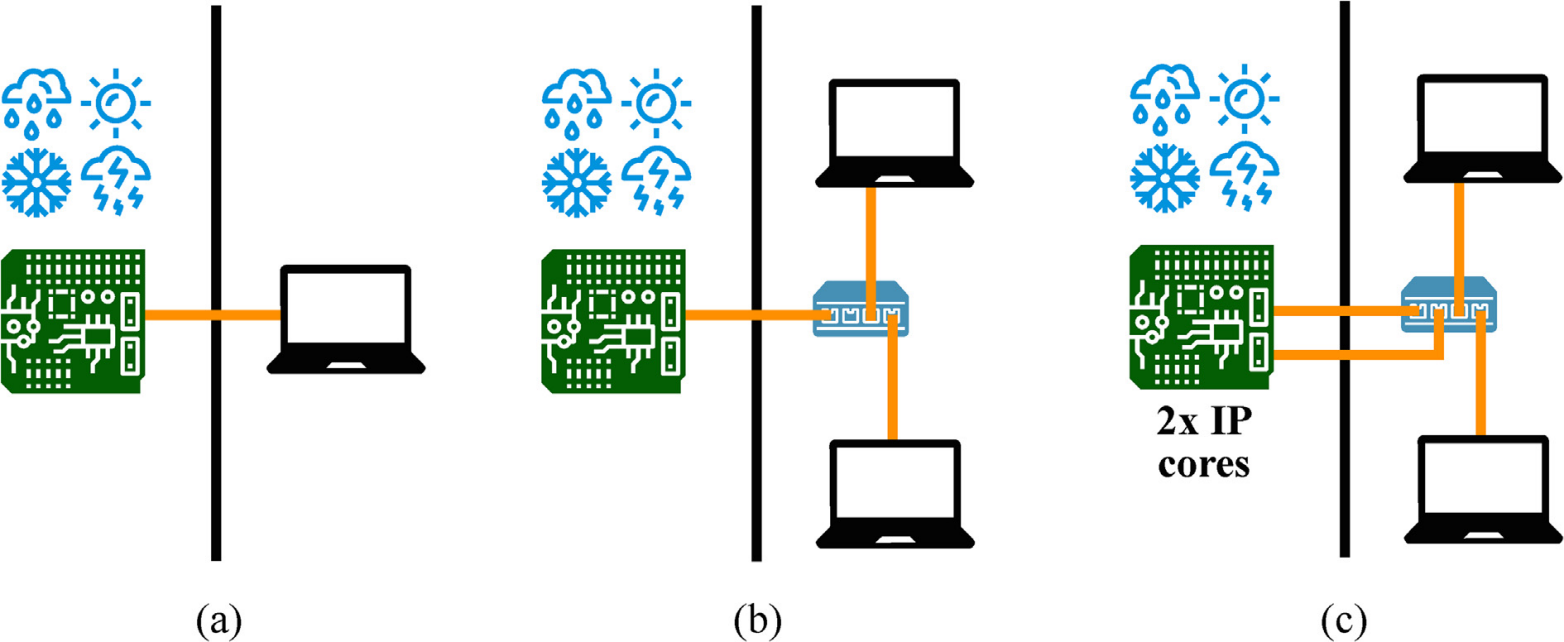
A possible way to increase reliability of the experimental setup. (a) Move PC to a safe area. (b) Use several PCs. (c) Provide full hardware and wire redundancy.

While carrying out long-term experiments, it is very important to ensure high reliability and to prevent the loss of collected data. The proposed design provides several options in this regard. First of all, Ethernet interface makes possible to move PC, which captures data, up to 100 m away from the experimental area and place it in a safe place with a redundant power source (Fig. 6). This is very important for the experimental studies performed outdoor or in aggressive environments because even if the logic analyzer board gets damaged, all the data it has measured will be transmitted to PC and not stuck in the internal memory of the analyzer.

The next step to enhance reliability of the experimental setup is to increase the number of PCs simultaneously capturing data from a logic analyzer (Fig. 6b). It can be done with the use of the common Ethernet switch as proposed FPGA firmware transmits standard UDP broadcast frames.

In order to reach still higher level of reliability, the proposed design can be easily replicated inside of one FPGA and be connected to different PCs with the use of independent Ethernet PHYs (Fig. 6c), taking into account its low area consumption. In this case, the system will also become tolerant to/withstand single cable damages.

To summarize the above written, the proposed logic analyzer design perfectly meets the requirements for long-term jitter monitoring. It is easily scalable, highly customizable and cross-platform, and it can be implemented with the use of low-cost hardware and a fully open-source toolchain.

Application areas:

• in laboratory and field experimental studies requiring long terms monitoring;
• as a starting set of IP cores and software for the custom application specific FPGA-based laboratory equipment;
• for educational purposes, explaining the way how modern communication protocols can be implemented in FPGA without the usage of proprietary vendor-specific IP cores.

## 3 Design files

### 3.1 FPGA IP cores

**Table:** 

Design filename	File type	Open source license	Location of the file
*crc32.v*	HDL code	Creative Commons Attributioann-ShareAlike license	http://doi.org/10.5281/zenodo.4034301 Should be placed in *cores* folder

*rmii*_*send*_*byte.v*	HDL code	Creative Commons Attribution-ShareAlike license	http://doi.org/10.5281/zenodo.4034301 Should be placed in *cores* folder

*rmii*_*send*_*byte*_*v2.v*	HDL code	Creative Commons Attribution-ShareAlike license	http://doi.org/10.5281/zenodo.4034301 Should be placed in *cores* folder

*udp*_*send.v*	HDL code	Creative Commons Attribution-ShareAlike license	http://doi.org/10.5281/zenodo.4034301 Should be placed in *cores* folder

*ws*_*log*_*ch.v*	HDL code	Creative Commons Attribution-ShareAlike license	http://doi.org/10.5281/zenodo.4034301 Should be placed in *cores* folder

*ws*_*log*_*max.v*	HDL code	Creative Commons Attribution-ShareAlike license	http://doi.org/10.5281/zenodo.4034301 Should be placed in *cores* folder

*ws*_*trigger*_*ch.v*	HDL code	Creative Commons Attribution-ShareAlike license	http://doi.org/10.5281/zenodo.4034301 Should be placed in *cores* folder

### 3.2 Top HDL files for reference designs and corresponding build scripts

**Table:** 

Design filename	File type	Open source license	Location of the file
*core*_*info*_*ice40.m*	Build script	Creative Commons Attribution-ShareAlike license	http://doi.org/10.5281/zenodo.4034301 Should be placed in *top-ice40* folder

*pins.pcf*	Pin assignment file	Creative Commons Attribution-ShareAlike license	http://doi.org/10.5281/zenodo.4034301 Should be placed in *top-ice40* folder

*project.scr*	Build script	Creative Commons Attribution-ShareAlike license	http://doi.org/10.5281/zenodo.4034301 Should be placed in *top-ice40* folder

*synth*_*ice40.sh*	Build script	Creative Commons Attribution-ShareAlike license	http://doi.org/10.5281/zenodo.4034301 Should be placed in *top-ice40* folder

*ws*_*logger*_*ice40.bit*	FPGA configuration file	Creative Commons Attribution-ShareAlike license	http://doi.org/10.5281/zenodo.4034301 Should be placed in *top-ice40* folder

*ws*_*logger*_*ice40.v*	Software	Creative Commons Attribution-ShareAlike license	http://doi.org/10.5281/zenodo.4034301 Should be placed in *top-ice40* folder

*core*_*info.m*	HDL code	Creative Commons Attribution-ShareAlike license	http://doi.org/10.5281/zenodo.4034301 Should be placed in *top-nexys4* folder

*pins.ucf*	Pin assignment file	Creative Commons Attribution-ShareAlike license	http://doi.org/10.5281/zenodo.4034301 Should be placed in *top-nexys4* folder

*ws*_*logger.bit*	FPGA configuration file	Creative Commons Attribution-ShareAlike license	http://doi.org/10.5281/zenodo.4034301 Should be placed in *top-nexys4* folder

*ws*_*logger.v*	HDL code	Creative Commons Attribution-ShareAlike license	http://doi.org/10.5281/zenodo.4034301 Should be placed in *top-nexys4* folder

### 3.3 Octave software for captured data processing

**Table:** 

Design filename	File type	Open source license	Location of the file
*logdump.txt*	Sample data	Creative Commons Attribution-ShareAlike license	http://doi.org/10.5281/zenodo.4034301 Should be placed in *octave* folder

*parse*_*log.m*	Software	Creative Commons Attribution-ShareAlike license	http://doi.org/10.5281/zenodo.4034301 Should be placed in *octave* folder

*parse*_*k12.m*	Software	Creative Commons Attribution-ShareAlike license	http://doi.org/10.5281/zenodo.4034301 Should be placed in *octave* folder

*parsepack.m*	Software	Creative Commons Attribution-ShareAlike license	http://doi.org/10.5281/zenodo.4034301 Should be placed in *octave* folder

### 3.4 Design file description

A brief description of the listed above design files is given in Table 1, Table 2, Table 3. And additional information about FPGA resource consumption of provided reference design can be found in Table 1. The proposed design has ultra-small area footprint and doesn’t require any dedicated memory or Digital Signal Processing (DSP-blocks), which make it compatible with the widest range of market-available FPGAs. HDL-related files listed in Table 2 contain full source code, required to build the projects or to modify it for your own needs. It is worth to mention, that proposed design doesn’t use any of proprietary or vendor-limited IP cores. Table 3 describes a set of Octave scripts, which serve as example, how the logic analyzer’s output data can be automatically processed into long-term jitter timing diagrams. This example is accompanied with sample data captured during experiment described in a Section 7.

**Table 1:** Pre-compiled FPGA firmware.

Design filename	Target	Resource consumption
*ws*_*logger*_*ice40.bit*	Lattice iCEstick kit (Lattice iCE40HX1K FPGA)	Logical cells: 988/ 1280
		Flip Flops: 598
		RAM blocks: 0/16

*ws*_*logger.bit*	Digilent Nexys 4 DDR kit (Xilinx XC7A100T FPGA)	Slices: 243/15850
		LUTs: 693/63400
		Flip Flops: 626/126800
		RAM blocks: 0/270
		DSP blocks: 0/240

**Table 2:** HDL-related design files.

Design filename	Target	Description
*crc32.v*	Common	Supplementary IP core used by **udp**_**send** module to evaluate Ethernet CRC-32 frame checksum.

*rmii*_*send*_*byte.v*	Common	Reduced Media Interface (RMII) for designs clocked at 100 MHz

*rmii*_*send*_*byte*_*v2.v*	Common	Reduced Media Interface (RMII) for designs clocked at 50 MHz

*udp*_*send.v*	Common	UDP sender IP Core

*ws*_*log*_*ch.v*	Common	IP core, which timestamps edges using time source provided by **ws**_**trigger**_**ch** module

*ws*_*log*_*max.v*	Common	IP core, which dynamically controls an output rate of the logical analyzer and evaluates maximum jitter value per defined time range.

*ws*_*trigger*_*ch.v*	Common	IP core, which detects edge on a master channel and creates a mutual time source for one another channel in a measurement window (Fig. 5).

*ws*_*logger*_*ice40.v*	Lattice iCEstick kit	2-channel logic analyzer reference design top module

*pins.pcf*	Lattice iCEstick kit	Pin assignment file for Lattice iCEstick evaluation kit

*core*_*info*_*ice40.m*	Lattice iCEstick kit	Design configuration file for COREbase automated building environment [23]

*synth*_*ice40.sh*	Lattice iCEstick kit	Bash build script for IceStorm [24] open-source FPGA toolchain

*project.scr*	Lattice iCEstick kit	Supplementary build script for Yosys [25]

*ws*_*logger.v*	Digilent Nexys 4 DDR kit	4-channel logic analyzer reference design top module

*pins.ucf*	Digilent Nexys 4 DDR kit	Pin assignment file for Digilent Nexys 4 DDR

*core*_*info.m*	Digilent Nexys 4 DDR kit	Design configuration file for COREbase automated building environment [23]

**Table 3:** Data processing scripts and sample date.

Design filename	Description
*logdump.txt*	Contains sample data captured by reference design on a basis of Digilent Nexys 4 DDR kit during clock jitter monitoring between four different devices.

*parse*_*log.m*	Example script, which shows how to parse *logdump.txt* sample data file with the **parse**_**k12** function and plot worst-case jitter timing diagrams.

*parse*_*k12.m*	Function, which parses frame capture files created with the use of a plain text K12 format, converts them to byte vectors and filter frames generated by devices other than the proposed logic analyzer.

*parsepack.m*	Supplementary function used by *parse*_*k12.m*.

## 4 Bill of materials

A total cost of the components depends on a number of channels and the chosen FPGA platform. The most easy-to-implement solution would be to buy one of the following Diligent FPGA kits: Nexys 4, Nexys 4 DDR (4-channel logic analyzer reference design for this kit is provided with the paper in *top-nexys4* folder) or Nexys A7. A new kit will cost 229–265 USD or even lower if one is able to get Diligent Academic discount. Meanwhile, the design is written on pure Verilog and can be synthesized nearly for any FPGA hardware architecture. In this case, in order to implement the proposed the logic analyzer the researcher will need to additionally purchase WaveShare LAN8720 ETH Board Ethernet communication module as well a few wires to connect it to FPGA Printed Circuit Board (PCB). The sample bill of materials for one of the cheapest possible configurations is provided below. As an additional feature, iCE40 FPGA, used in this configuration, can be programmed with open-source software [26] provided by IceStorm project [24], which make this design fully proprietary-free in terms of both hardware and software. The 2-channel logic analyzer reference design as well as build scripts for the open toolchain are provided with this paper in *top-ice40* folder.

**Table:** 

Designator	Component	Number	Cost per unit currency	Total cost	Source of materials	Material type
Lattice Semiconductor	ICE40HX1K-STICK-EVN	1	49.5 USD	49.5 USD	Lattice Semiconductor portal	semi-conductor

WireShare	LAN8720 ETH Board	1	9 USD	9 USD	WireShare portal	semi-conductor

–	Solderless Flexible Breadboard Wires	6	0.5 USD	3 USD	Amazon	metal

## 5 Build instructions

### 5.1 Hardware wiring

Hardware wiring depends on the PCB board used to implement logic analyzer. The paper is provided with 2 reference designs: one is based on Digilent Nexys 4 DDR and the second is based on low-cost Lattice iCEstick kit. Via the example of these two development kits it will be shown how to implement and configure logic analyzer for long-term jitter monitoring. Finally, it is possible to implement the proposed design nearly on any kind of FPGA kit in the market introducing minimal changes to the above mentioned reference designs.

The design requires FPGA, which is clocked at 50 or 100 MHz and is connected to at least 2 digital inputs dedicated for measurement channels, one LED and Ethernet PHY with RMII. It is highly recommended to use Microchip Technology LAN8720A Ethernet PHY, as all the tests carried out in the paper were performed with this integrated circuit.

The subject matter building device on a basis on Digilent Nexys 4 DDR kit is an easy task, as it already has everything onboard. It is worth mentioning that after a brief documentation analysis it seems that the newer kit Digilent Nexys A7 is fully compatible with the design provided with the paper for Digilent Nexys 4 DDR without any additional changes (unfortunately, the Author didn’t have a chance to test it himself).

**Fig. 7:**
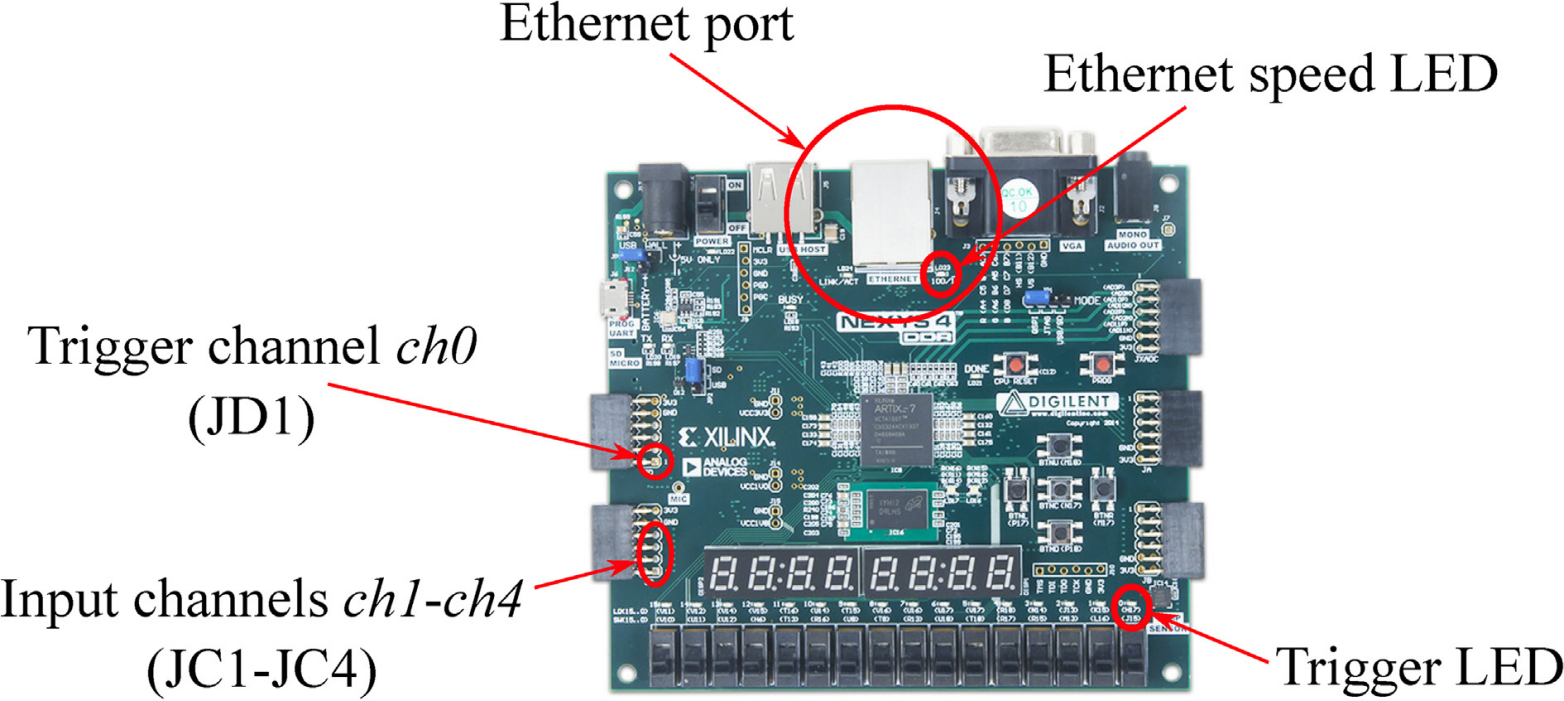
Logic analyzer on a basis of Digilent Nexys 4 DDR board.

The location of the main logic analyzer elements when implemented on a basis of Nexys 4 DDR kit is shown in Fig. 7.

In difference to the previous example, Lattice iCEstick kit will require additional wiring because it is not equipped neither with Ethernet PHY nor with a proper clock generator. Fortunately, both of these problems can be solved with one WireShare LAN8720 ETH Board, which should be connected to the Lattice iCEstick board according the Table 4.

**Table 4:** Logic analyzer pin description.

iCEstick Pmod pin	LAN8720 ETH Board pin	Logic analyzer
1	TX_EN	Ethernet
2	TX1	Ethernet
3	–	Channel *ch0*
4	–	Channel *ch2*
5	–	GND
6		+3.3 V
7	TX0	Ethernet
8	NC	–
9	–	Channel *ch1*
10	nINT/RETCLK	50 MHz clock from PHY
11	GND	Ethernet power supply
12	VCC	Ethernet power supply

**Fig. 8:**
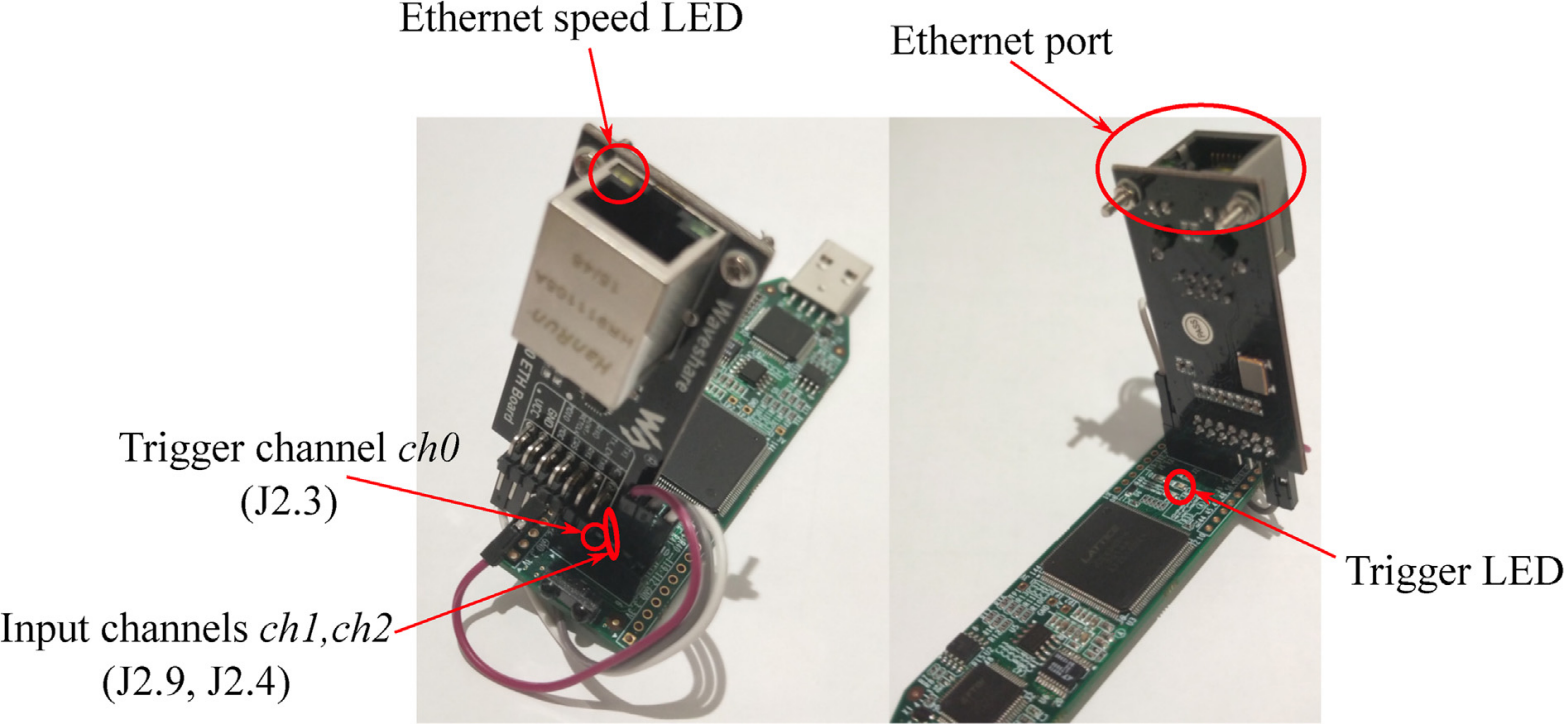
Logic analyzer on a basis of Lattice iCEstick.

In order to reduce the number of the wires, WireShare LAN8720 ETH Board can be partially inserted in a Pmod connector as it is shown in Fig. 8. In this case REFCLK pin of WireShare board will point directly to the Lattice iCEstick PCB, so this pin should be bent a little bit to fit a solderless wire connector.

Adopting proposed design to other FPGA kits will require changing Pin assignment files to make them compliant with kit schematics. If the clock generator of the kit is not 50 or 100 MHz, then, one of these frequencies can be usually synthesized with the use of FPGA PLL module, but as far as it is crucial for Ethernet interface to have one of those frequencies, it is always better to have a proper clock generator onboard or to use WireShare LAN8720 ETH Board kit, which is already equipped with it.

### 5.2 Uploading FPGA firmware

The easiest way to run a reference design provided with this paper is to upload FPGA with one of the precompiled firmware file. In order to do this for Digilent Nexys 4 DDR, you will need to use *ws*_*logger.bit* and follow instructions in Section 3 of the corresponding vendor’s guide [27]. Programming iCEstick kit is possible with the use of open-source tool **iceprog** from IceStorm project [24] project. Just go to *top-ice40* folder and run:

**iceprog ws**_**logger**_**ice40.bit**

### 5.3 Building FPGA firmware

There are several options to build FPGA firmware from the source. Before using any of them you should check that the file structure and folder names are the same as described in the Section 3 tables.

If you are using COREbase automated building environment [23], you can simply go to the folder with the corresponding reference design and call the **build**_**core** function. Necessary *core*_*info* files with the right design file structure and chip information are already stored in the folders with top HDL modules.

Building FPGA firmware manually is not much more difficult than using COREbase. One will need to add all HDL files from the *cores* and *top-nexys4* folders into one’s favorite Xilinx tool (Vivado or ISE), set *ws*_*logger.v* as top file, include *pins.ucf* as User Constraints File and define XC7A100T-1CSG324C as target FPGA. Then one can build firmware according to Xilinx guide to the chosen software.

In order to build FPGA firmware for iCEstick, one can use official Lattice iCE Cube 2 software [28] and follow the same procedure as it has been described above for Xilinx, but using files from *top-ice40* folder instead of using the ones from *top-nexys4*. The other alternative is to install IceStorm tools [24] and run *synth*_*ice40.sh* script inside *top-ice40* folder. The script will build FPGA bitstream file using fully open-source IceStorm toolchain.

### 5.4 Modifying FPGA firmware

As it has been mention before the logic analyzer proposed in the present paper is not a stand-alone device with final functionality, but rather a set of area-efficient cross-platform IP cores, which can be used to build an application specific tool with minimum additional efforts. This section describes how these IP cores can be configured and customized to achieve the desired functionality.

**Fig. 9:**
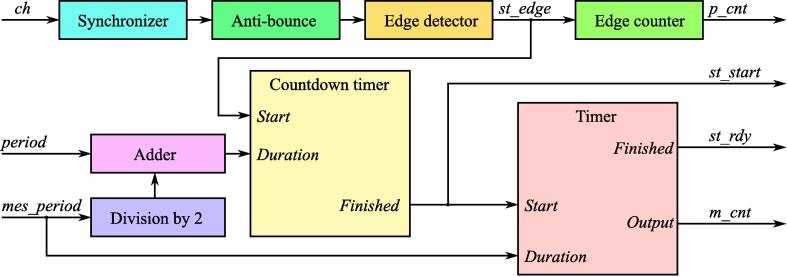
ws_trigger_ch IP core block diagram.

Any configuration of the logic analyzer should have one trigger signal. In the provided reference designs it is always *ch0*. The signal should be passed to *ch* input of **ws**_**trigger**_**ch** IP core (Fig. 9), which will generate a set of output signals required for edge capturing and timestamping according the algorithm described in Section 2 (Fig. 5):

• *st*_*start* – strobe indicating start of the acquisition period;
• *st*_*rdy* – strobe indicating end of the acquisition period;
• *m*_*cnt* – cycle counter, which starts after *st*_*start* and counts before *st*_*rdy*;
• *p*_*cnt* – counter of trigger events.

**ws**_**trigger**_**ch** has two main timing parameters passed as module inputs:

• *period* – the expected number of clock cycles between *ch* input edges (*Tp*).
• *mes*_*period* – the number of clock cycles between *st*_*start* and *st*_*rdy* (*Tm*).

If input signal is periodic with period close to 2·periods, *st*_*start* will be generated $\frac{\mathrm{mes}\_\mathrm{period}}{2}$ cycles before *ch* edge and *st*_*rdy* will be generated $\frac{\mathrm{mes}\_\mathrm{period}}{2}$ cycles after it (Fig. 5).

Also module **ws**_**trigger**_**ch** has 4 parameters, each of which should be predefined before build procedure:

• *Np* – bit width of *period* input;
• *Nm* – bit width of *mes*_*period* input;
• *Nc* – bit width of *p*_*cnt* counter;
• *Na* – the number of clock cycles during which *ch* input shouldn’t change its level after each edge (used as filter to prevent multiple edges caused by electromagnetic interference).

**Fig. 10:**
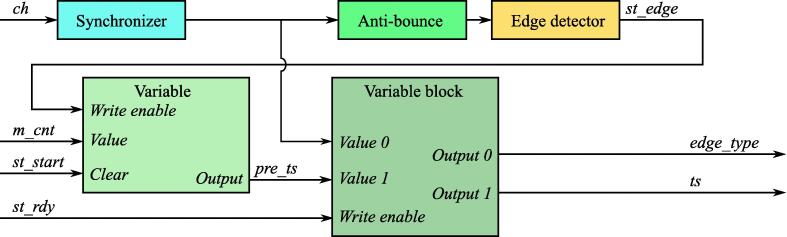
ws_log_ch IP core block diagram.

In order to timestamp signal edges occurred during acquisition period for each signal, including the one acting as trigger, a **ws**_**log**_**ch** (Fig. 10) should be used. It takes input channel signal level and **ws**_**trigger**_**ch** outputs as its inputs and results two values:

• *edge*_*type* – type of the timestamped edge (0 – negative, 1 – positive);
• *ts* – timestamp of the edge in clock cycles starting from *st*_*start*.

It should be mentioned that both of these outputs are generated on the next cycle after *st*_*rdy*, but not syncrhonously with it.

**ws**_**log**_**ch** has 2 parameters, *Nm* and *Na*, which has the same meaning as for **ws**_**trigger**_**ch** module.

The output timestamp *ts* of each **ws**_**log**_**ch** module, excluding the one processing signal used as trigger, is driven to a *ts* input of the separate **ws**_**log**_**max** module. *tr* input of all **ws**_**log**_**max** modules is connected to *ts* output of **ws**_**log**_**ch** module, which is a processing trigger signal. The other 2 inputs, *st*_*rdy* and *prescaler* of each **ws**_**log**_**max** modules are connected to *st*_*rdy* and *st*_*pcnt* outputs of **ws**_**trigger**_**ch** modules respectively.

The main aim of **ws**_**log**_**max** modules is to evaluate the jitter value (*jtr* output) for each input signal and generate strobe for UDP processing IP core to transmit a new jitter value to PC. At the same time the module can dynamically change Ethernet exchange rate, which can be configured by two parameters *Npr* and *Nl*. The last parameter *Nm* should be the same as for the corresponding **ws**_**log**_**ch** module.

**Fig. 11:**
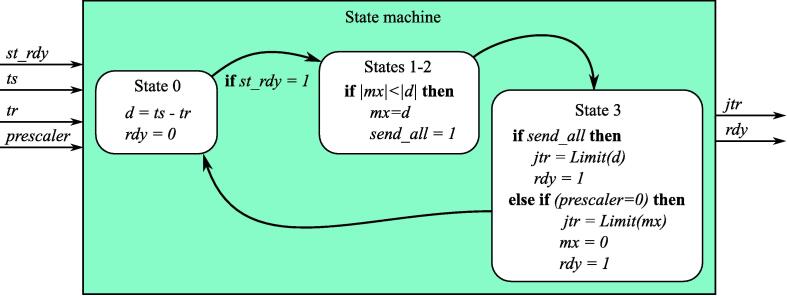
ws_log_max IP core block diagram.

**ws**_**log**_**max** (Fig. 11) module evaluates jitter as difference between *ts* and *tr* edge timestamps accordingly (1).

(1) jtr=ts-tr

If the condition (2) is met, the output value of the module will be updated every $2^{\mathrm{Npr}}$ measurements, and it will be equal to the *jtr* with maximum absolute value evaluated during the last $2^{\mathrm{Npr}}$ measurements. If condition (2) is not met, *jtr* output will be updated after each measurements until *Npr* lower bits of *prescaler* input will not reach zero. Each time *jtr* output changes, *rdy* strobe is generated on a **ws**_**log**_**max** module output to trigger outgoing UDP transmission.

(2) $$-2^{\mathrm{Nm}-\mathrm{Nl}+1}-1<|\mathrm{jtr}|<2^{\mathrm{Nm}-\mathrm{Nl}+1}$$

Thus, if jitter values are below the limits defined by (2), the logic analyzer switches to a low exchange data rate and sends the worst-case jitter between transmissions. On the contrary, if jitter raises beyond the limit, it is considered as point of interest and the logic analyzer starts to send each single measurement to PC.

**ws**_**log**_**max** is a simple example how input signal preprocessing with a variable data exchange rate can be implemented for a specific application of long-term jitter analysis. Using such kind of modules is an effective way to optimize the trade-off between the amount of the data collected during a long experiment and enough detail level around specific points of interest.

Finally, as all the measurement channels are totally independent, then in order to increase their number a reference design should be modified in a following way:

• Declare ts,jtr and *rdy* wires corresponding to additional channels in the beginning of the top module.
• Add a set of **ws**_**log**_**ch**, **ws**_**log**_**max** for each new channel and connect them in the same way it is done for the channels already existing in the reference design.
• Add *rdy* output signals of the new **ws**_**log**_**max** modules to the OR concatenation with *rdy* signals from other **ws**_**log**_**max** modules.
• Add *jtr* values evaluated for the new signals to a UDP payload.

The clarification how to configure UDP transmissions parameters, including frame payload, will be provided in the next subsection.

Ethernet communication as a part of design was created in a such a way to make it both easy to configure and compatible with the most FPGAs available on the market. Generally, it consists of two modules and a payload memory block.

The main part, which shall be configured by the end-user is a distributed memory block described in one of the reference designs as:

**Table:** 

reg [7:0]payload[0:17];
always @(*)
begin
payload[0] = pcnt[7:0];
payload[1] = pcnt[15:8];
payload[2] = pcnt[23:16];
payload[3] = pcnt[31:24];
payload[4] = jtr1[7:0];
payload[5] = jtr1[15:8];
payload[6] = jtr2[7:0];
payload[7] = jtr2[15:8];
payload[8] = jtr3[7:0];
payload[9] = jtr3[15:8];
payload[10] = 0;
payload[11] = 0;
payload[12] = 0;
payload[13] = 0;
payload[14] = 0;
payload[15] = 0;
payload[16] = 0;
payload[17] = 0;
end

The first line of the above listing defines memory, which consists of 18 cells of 8 bits each. Such kind of memory will not be generated in a memory block during synthesis but will be used as a simple user interface to define each byte of a payload in UDP frames transmitted by the logic analyzer. If more data is needed, then the size of memory can be increased from 18 to a bigger number of cells. The maximum size of the payload is limited by Maximum Transmission Unit (MTU) parameter of the PC Ethernet interface. Usually MTU is 1500, so the maximum size of the payload will be 1454 bytes. It is also important that the payload memory size should never be less than 18 because in this case resulting UDP frames will be less than 64 bytes and thus can be declined by Ethernet interfaces of some PCs. If some part of payload is unused, it is better to force these bytes to zero in the same the way it is done in the provided reference example.

The next “**always**” statement defines the values that will be stored in each byte of UDP payload. It is worth mentioning that the bytes in the *payload* memory are addressed from 0, but when corresponding frames will be parsed in Wireshark capture results zero byte of payload will become 43rd byte of captured frame as the first 42 bytes store service information dedicated to Ethernet and UDP communication protocols.

On a basis of defined payload **udp**_**send** IP core generates UDP frame with structure provided in Table 5. It should be noted, that according current standards most frames fields dedicated to Ethernet and IP headers are big-endian, while Ethernet checksum is little-endian. Logic analyzer measurements transferred as UDP payload are also little-endian, but generally byte order of this data can be change in any way by modifying “**always**” statement, which defines content of *payload* memory.

**Table 5:** UDP frame structure generated by udp_send IP core.

Frame bytes	Value	Field description
0–7	0xD555 5555 5555 5555	Ethernet preamble
8–13	*dst*_*addr*	Destination MAC address (MAC address of the PC)
14–19	*src*_*addr*	Source MAC address (MAC address of the logical analyzer)
20–21	0x08 00	Protocol type – IPv4
22–23	0x45 00	IP protocol version 4 with 20 bytes header size
23–24	28+p_sz	Datagram length
26–29	0x0000 0000	IPv4 Service fields
30	0x40	Datagram time to live (TTL)
31	0x11	IP protocol type – UDP
32–33	*ip*_*crc*	IP header checksum
34–37	*src*_*ip*	Source IP address (IP address of the logical analyzer)
38–41	*dst*_*ip*	Destination IP address (IP address of the PC)
42–43	*src*_*port*	Source port
44–45	*dst*_*port*	Destination port
46–47	8+p_sz	UDP length
48–49	0x0000	UDP checksum. 0x0000 means, that checksum should be ignored
50-(50+p_sz)	payload[0..(p_sz-1)]	Frame payload
(51+p_sz) – (54+p_sz)	*eth*_*crc*	Ethernet checksum

**udp**_**send** IP core has several parameters that should be configured before use:

• *dst*_*addr* – MAC address of the destination device. By default, it is set to broadcast, but can be changed to MAC address of the PC to use unicast transmissions. The use of broadcast makes possible to receive the frames by any software on any computer, without rebuilding FPGA firmware.
• *src*_*addr* – MAC address of the logic analyzer itself. If the logic analyzer has point-to-point connection with PC, it can take any value different from the PC MAC address.
• *dst*_*ip* – IP address of the PC. By default, it is set to broadcast *192.168.0.255*, and in case of point-to-point connection with PC it should work with any address as Wireshark performs capturing on a pure Ethernet layer. Meanwhile, in case of using switches between PC and the logic analyzer the IP address should be in the same subnetwork as PC. In case of using Wi-Fi segment to connect logic analyzer, it is only IP address of the PC that should be used (broadcast transmissions should be avoided).
• *src*_*ip* – IP address of the logic analyzer. It should be any free address in the network.
• *dst*_*port* – UDP port used for transmissions.
• *src*_*port* – UDP port used for transmissions.
• *p*_*sz* – Size of *payload* memory in bytes.
• *Nsz* – frame byte counter bit width. It should be chosen as ceil round value of $\log_{2}(p\_\mathrm{sz}+55)$

Address signal used to access *payload* memory in line:

.payload(payload[addr[4:0]]),should be shrinked to $\mathrm{ceil}(\log_{2}p\_\mathrm{sz})$ to ensure correct synthesis.

**udp**_**send** module uses three signals: *tx*_*start*, *tx*_*data* and *tx*_*rdy*, to communicate with RMII transmitter module. There are two versions of RMII transmitters provided with the paper.

**rmii**_**send**_**byte** – should be used on the devices clocked at 100 MHz. The example of its usage is provided in the reference design for Digilent Nexys 4 DDR kit.

**rmii**_**send**_**byte**_**v2** – should be used on the devices clocked at 50 MHz. The example of its usage is provided in the reference design for iCEstick.

It is worth mentioning that in case of **rmii**_**send**_**byte**_**v2** module usage on FPGA kits, where RMII clock is generated by FPGA (such as Digilent Nexys 4 DDR), this clock should have phase shift of 45, 90 or even 180 degree relative *clk* to compensate clock skew introduced by FPGA logic, used for its generation [29]. This phase shift can be achieved by using PLL or in some cases simple logical NOT operation can be used to create 180 phase shift.

Generally, both versions of the provided RMII transmitter IP cores do not require any tuning and should be used in the same way as it is demonstrated in the reference design. The only input that can be changed is *fast*_*eth*. By default, it is set to 1, which corresponds to Fast Ethernet (100 Mbps). In some cases, the project limitations may require switching to the lower speed of 10 Mbps. In this case, this parameter should be changed by zero.

One should be aware that changing of RMII transmitter speed does not automatically imply that PHY switching to a lower speed itself. The speed for LAN8720A used in the provided reference designs can be changed by setting MODE pins during initialization procedure. It is important to mention that even these pins are not used for communication in the current logic analyzer design. During regular operation they act as input signals from the PHY receiver, which means that connecting them to any voltage level permanently may cause electrical damage to the PHY or FPGA. In order to do initialization correctly, the pins should be configured as *inout* and switched to Z-state immediately after PHY reset switches to a high level. The example of such speed initialization is demonstrated in the reference design for Digilent Nexys 4 DDR kit, where PHY is switched to speed detection with the use of auto-negotiation. For information on other MODE states corresponding to a specific speed please refer to PHY manual.

## 6 Operation instructions

**Fig. 12:**
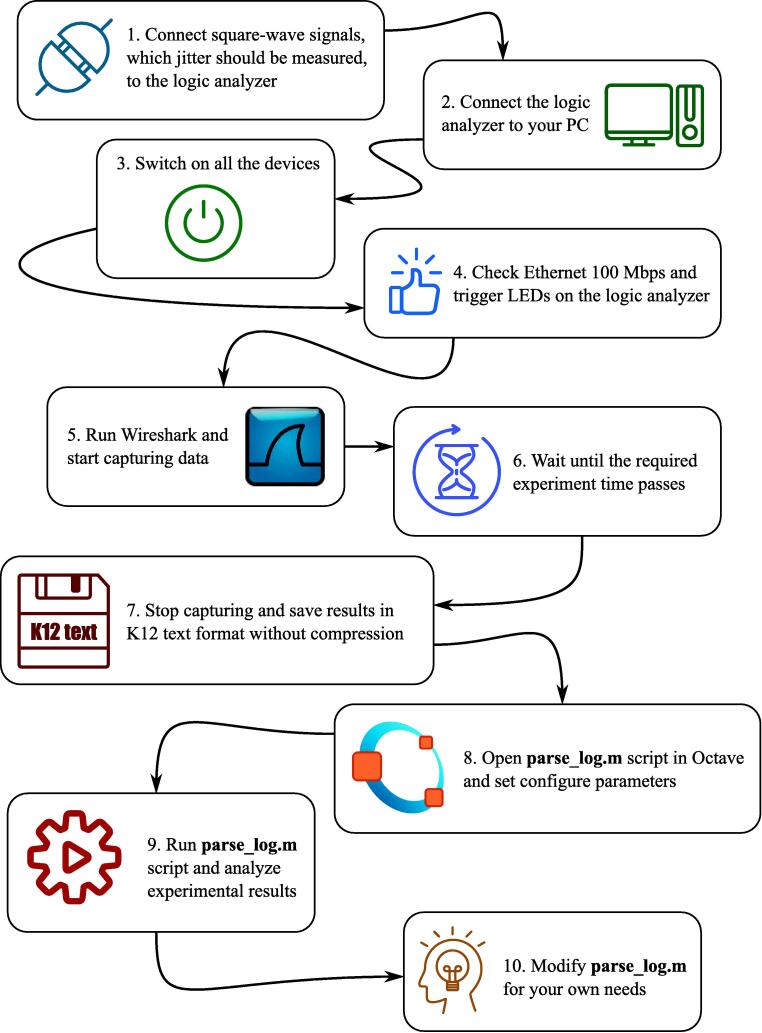
Flow chart of main operation procedures.

**Fig. 13:**
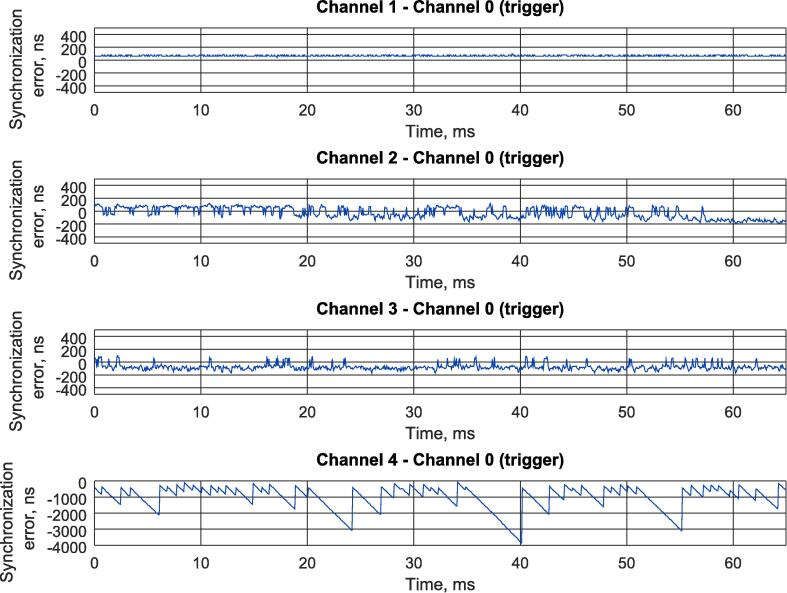
The result of parsing experimental data from the capture file provided with the paper (see Section 7 for details of the experiment).

A sequence of main operation procedures is shown on Fig. 12. A detailed description of each of them can be found in a list below:

• Connect external devices with their ports, which generate 1 kHz square wave, to input ports of the logic analyzer. Remember to connect ground pins of all the devices to a ground pin of the logic analyzer. The instruction generally assumes that all the input signals are 3.3 LVCMOS. Digital signals of other types may need additional hardware or/and additional configuration in synthesis tools before building FPGA hardware. If external devices generate square wave with the frequency different to 1 kHz, you will need to rebuild FPGA firmware following instructions in Section 5.
• Connect logic analyzer to Ethernet interface of the PC.
• Disable all protocols in Ethernet interface settings of your PC to prevent generating of the additional traffic in Wireshark capture. Generally, the proposed solution is very robust to any additional traffic, so it should not affect the measurements but will only enlarge the overall size of file with experimental data.
• Switch on all devices.
• Check 100 Mbps LED on Ethernet PHY (Fig. 7, Fig. 8). If it is off, then auto-negotiation algorithms switched the Ethernet speed to 10 Mbps. Try to change Ethernet cable. If you are using FPGA kit different from the one used in the reference designs provided with the paper, then check if MODE pins have proper state during PHY initialization and do not switch off auto-negotiation. Finally, if you are not able to reach 100 Mbps mode, you can reduce Ethernet speed to 10 Mbps and rebuild FPGA firmware following instructions in Section 5.
• Check trigger LED blinking. If it is not, check Channel 0 (*ch0*) wiring of the logic analyzer.
• Run Wireshark and start capturing Ethernet interface. Check if the number of captured packets displayed in a status bar is increasing.
• Stop capturing when it is necessary.
• Save captured data as “K12 text file” without compression.
• Run octave.
• Open *parse*_*log.m*.
• Set first parameter of the **parse**_**k12** function to a filename of the saved K12 file with experimental data.
• Set second parameter of the **parse**_**k12** function to a number of captured frames saved in K12 file with experimental data. This parameter is used only to optimize memory management operations and reduce the overall Octave script runtime. Generally, it is better to set the value bigger or equal to a number of captured frame because each frame parsed after the number defined in the parameter will cause memory reallocation. If processing time is not important, the parameter can be set to 1.
• Set the third parameter of the **parse**_**k12** function to a MAC address of the logic analyzer predefined in FPGA firmware during build procedure (see Section 5). The parameter should be a vertical vector of 6 elements corresponding to each byte of MAC address.
• Set the forth parameter of the **parse**_**k12** function to an IP address of the logic analyzer predefined in FPGA firmware during build procedure (see Section 5). The parameter should be a vertical vector of 4 elements, corresponding to each byte of IP address.
• Set **ts** variable to 10 if the logic analyzer is clocked at 100 MHz (Nexys 4 DDR kit) or to 5 if it is clocked at 50 MHz (iCEstick kit).
• Set the master clock square wave frequency in **freq** variable to plot timing diagrams with the right scale.
• If UDP frame payload was changed in FPGA firmware, then modify corresponding “*for*” cycle according to new frame structure (see Section 5).
• Run *parse*_*log.m*. If everything was done correctly, the data collected during the experiment will be displayed on the screen. If you use sample experimental data from **logdump.txt** file, the output results will look the same as in Fig. 13, Fig. 16. Of course, *parse*_*log.m* is only an example how a script for processing of data captured with proposed logic analyzer can look like. It can be modified in different ways during real experimental studies to provide automated result analysis and its demonstration in the most convenient way.

## 7 Validation and characterization

**Fig. 14:**
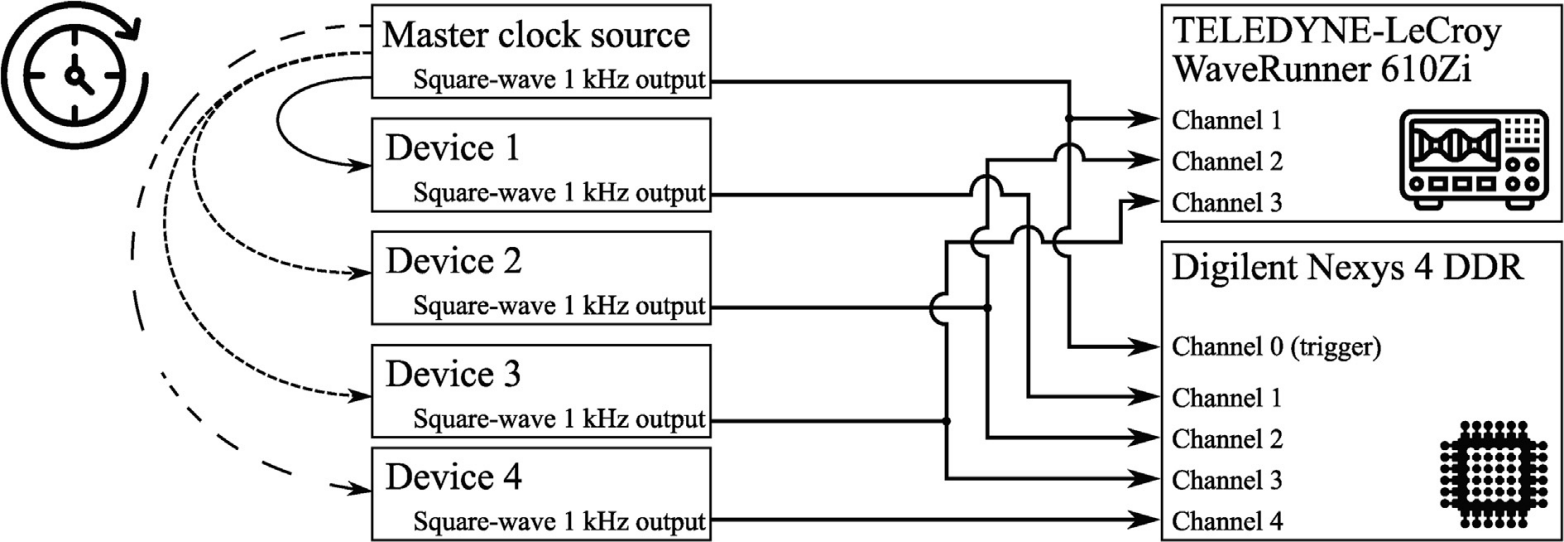
Testbench structure.

In order to verify the functionality of the proposed logic analyzer, a testbench was built (Fig. 14). It consists of 4 devices synchronized to mutual master clock by 3 different methods: Device 1 by the most precise one, Devices 2 and 3 – less precise and Device 4 by the least precise method characterized by an unstable synchronization period. The master clock generator and all the slave devices generated 1 kHz square-wave signals were synchronized to their internal clock. The signals were connected to the 4-channel logic analyzer reference design provided with the paper. Square waves synchronous to the master clock generator and Devices 2, 3 were also connected to the digital oscilloscope TELEDYNE-LeCroy WaveRunner 610Zi. This oscilloscope was working in a Persistence mode with synchronization on both edges of the trigger channel connected to the master clock generator. The oscilloscope screen was cleared simultaneously with the start of capturing data from the logic analyzer in Wireshark, and the data acquisition on oscilloscope was stopped immediately after stopping capturing in Wireshark. The data captured during the experiment is provided with the paper in *logdump.txt* as a sample dataset for testing Octave-based software.

**Fig. 15:**
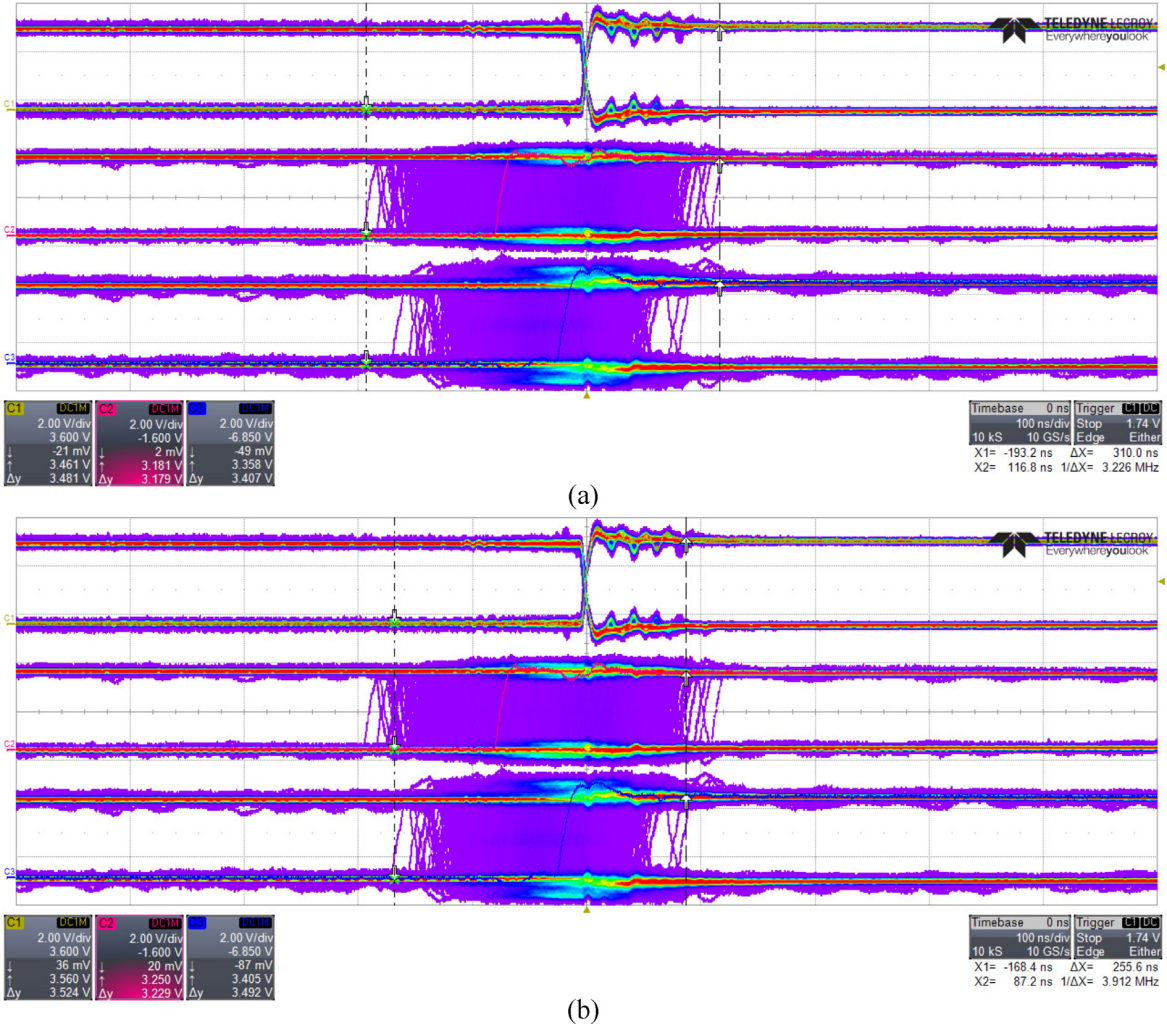
The screenshot of the jitter measurements evaluated with the digital oscilloscope working Persistence mode. (a) Device 2-Master clock jitter measurement. (b) Device 3-Master clock jitter measurement.

After data acquisition was stopped, the maximum jitter was measured with the use of the oscilloscope cursors tool (Fig. 15). The top oscillogram (C1) on (Fig. 15) is a reference square wave generated on a basis of master clock and captured on both rising and falling edges. Middle (C2) and bottom (C3) oscillograms correspond to Device 2 and Device 3 respectively. As it can be seen their edges have variable time shift to the edges of the reference C1 channel. The total range of those variations correspond to the synchronization jitter of each of the devices. Part (a) of the Fig. 15 shows Device 2 jitter measurement, while part (b) is dedicated to measuring of Device 3 jitter.

**Fig. 16:**
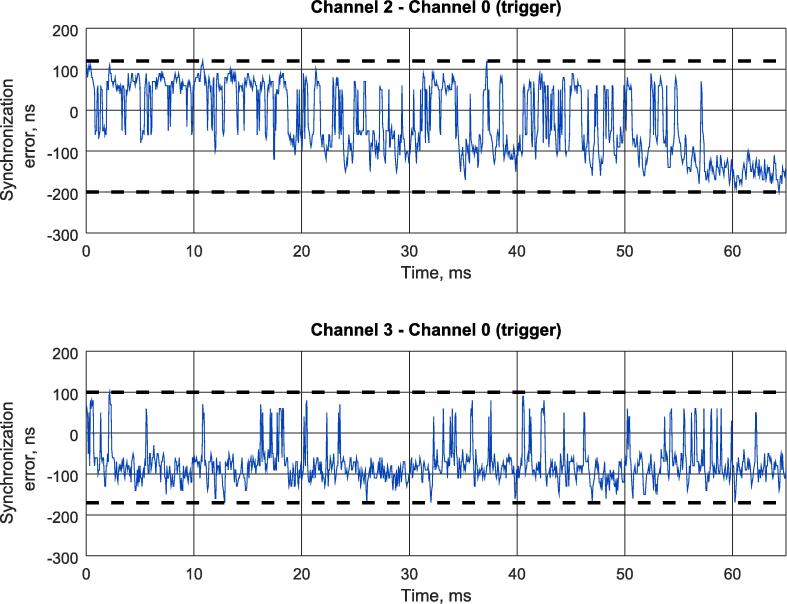
The immediate jitter evaluated with the logic analyzer. (a) Device 2-Master Clock. (b) Device 3-Master clock.

The data captured with Wireshark was processed with *parse*_*log.m* Octave script provided with the paper. The timing diagrams of the measurements for all 4 input channels of the logic analyzer are shown in Fig. 13. The measurements corresponding to Devices 2 and 3 were additionally displayed on separate plot window (Fig. 16), while their minimum and maximum values were evaluated using standard Octave functionality and printed in a console.

For sake of convenience, minimum and maximum values of immediate jitter evaluation with different methods are summarized in the Table 6.

**Table 6:** Experimental results

Parameter	Digital oscilloscope	Proposed logic analyzer	Absolute difference
Device 2 – Master minimum immediate jitter	−193.2 ns	−200 ns	6.8 ns
Device 2 – Master maximum immediate jitter	116.8 ns	120 ns	3.2 ns
Device 2 – Master jitter	310 ns	320 ns	10 ns
Device 3 – Master minimum immediate jitter	−168.4 ns	−170 ns	1.6 ns
Device 3 – Master maximum immediate jitter	87.2 ns	100 ns	12.8 ns
Device 3 – Master jitter	255.6 ns	270 ns	14.4 ns

Theoretically, edge timestamping precision of the logic analyzer running at 100 MHz is ±10 ns. The proposed logic analyzer evaluates the immediate jitter values as the difference between edge timestamps of input and reference channels, so total theoretical error can vary ±20 ns If we look at the achieved experimental results (Table 6), the differences between oscilloscope and the logic analyzer measurements perfectly fit the above mentioned theoretical limits. Finally, experimental result shows that proposed logical analyzer provides 20 ns precision which is similar to the one achieved by industrial real-time network analyzers such as B&R X20ET8819 [6] and is suitable for most tasks in real-time control and communication.

## Declaration of Competing Interest

The authors declare that they have no known competing financial interests or personal relationships that could have appeared to influence the work reported in this paper.
